# A Potential Link between Myeloperoxidase Modified LDL, Atherosclerosis and Depression

**DOI:** 10.3390/ijms25168805

**Published:** 2024-08-13

**Authors:** Jalil Daher

**Affiliations:** Department of Biology, Faculty of Arts and Sciences, University of Balamand, El-Koura P.O. Box 100, Lebanon; jalil.daher@balamand.edu.lb; Tel.: +961-6930250 (ext. 3718)

**Keywords:** atherosclerosis, Mox-LDL, depression, neuroserpin, tPA, BDNF, CD, MDD

## Abstract

Atherosclerosis is a chronic inflammatory disease that involves modified low-density lipoproteins (LDL) which play a pivotal role in the initiation and progression of the disease. Myeloperoxidase oxidized LDL (Mox-LDL) is considered to be the most patho-physiologically relevant type of modified LDL and has been reported to be ubiquitously present in atheroma plaques of patients with atherosclerosis. Besides its involvement in the latter disease state, Mox-LDL has also been shown to be implicated in the pathogenesis of various illnesses including sleep disorders, which are in turn associated with heart disease and depression in many intricate ways. Meanwhile, we have recently shown that lox-1-mediated Mox-LDL signaling modulates neuroserpin activity in endothelial cells, which could have major implications that go beyond the pathophysiology of stroke and cerebrovascular disease (CD). Of note is that tissue plasminogen activator (tPA), which is the main target of neuroserpin in the brain, has a crucial function in the processing of brain-derived neurotrophic factor (BDNF) into its mature form. This factor is known to be involved in major depressive disorder (MDD) development and pathogenesis. Since tPA is more conventionally recognized as being involved in fibrinolytic mechanisms, and its effect on the BDNF system in the context of MDD is still not extensively studied, we speculate that any Mox-LDL-driven change in the activity of tPA in patients with atherosclerosis may lead to a decrease in the production of mature BDNF, resulting in impaired neural plasticity and depression. Deciphering the mechanisms of interaction between those factors could help in better understanding the potentially overlapping pathological mechanisms that regulate disease processes in CD and MDD, supporting the possibility of novel and common therapeutic opportunities for millions of patients worldwide.

Atherosclerosis is a chronic inflammatory disease that involves vascular endothelial cells and immune cells, including macrophages, which are considered to be the hallmark of the disease. One of the major factors that is associated with the process of atherosclerosis is modified low-density lipoprotein (LDL) [1]. During the progression of the disease, modified LDL is recognized by macrophages and engulfed through their scavenger receptors, leading to the formation of foam cells (lipid-laden macrophages) which accumulate in the subendothelial space of arteries and contribute to the formation of an atheroma plaque [2]. Although several mechanisms have been proposed to describe the modification/oxidation of LDL particles, LDL that is modified by myeloperoxidase (MPO) remains the most patho-physiologically relevant type of oxidized LDL [3]. MPO is a member of a group of enzymes called heme peroxidases which are found in many organisms including mammals, fungi and plants [4]. These enzymes are endowed with the capacity to oxidize numerous molecules, including halides, through hydrogen peroxide (H_2_O_2_)-mediated reactions. As far as MPO is concerned, it is mainly stored in the azurophilic granules of neutrophils and is released into the phagosomes of activated cells during oxidative burst, where it is responsible for the conversion of H_2_O_2_ into hypochlorous acid (HOCl), which is considered to be one of the most powerful oxidants secreted in the human body [4,5,6]. While MPO is considered as a pivotal element of the innate branch of the immune system due to its bactericidal activity, it has been also linked to multiple pathologies, including atherosclerosis [6]. Thus, MPO is recognized as a major player in health and disease and several studies have been advertising it as a potential biomarker for cardiovascular diseases (CVDs) because of its ubiquitous presence in atheroma plaques where it modifies LDL into its oxidized form, leading to the initiation of inflammatory pathways and affecting the progression of the atherosclerotic disease [2]. In the context of inflammation, MPO has been associated with the pathogenesis of numerous diseases which are all linked to either acute or chronic inflammatory conditions. While MPO effects are beneficial when the body is fighting infections, MPO-derived oxidant species are detrimental to the tissues because of their interactions with their various components including lipids, proteins and nucleic acids [7]. Thus, MPO has been tightly linked to the pathogenesis of several types of cancer such as lung, colon, breast, bladder, larynx and stomach cancer [8]. Also, in the context of neurodegenerative diseases, MPO has been closely associated with multiple pathological processes that lead to Parkinson’s and Alzheimer’s disease [9]. Further reports have also shown that MPO could play a role in the progression of major inflammatory disorders including rheumatoid arthritis, vasculitis, pancreatitis, periodontitis and inflammatory bowel disease [7]. Finally, the main MPO byproduct, MPO-oxidized LDL (Mox-LDL), has been reported to be involved in the pathogenesis of several important disease states that are related to kidney inflammation and failure, erectile dysfunction and sleep disorders [2]. Given the fact that the latter disorders have been shown to be associated with atherosclerosis and mental health problems including depression [10,11,12], establishing a link that bridges Mox-LDL, atherosclerosis and depression is particularly relevant to the present article.

We have previously reported a proinflammatory role of Mox-LDL that correlates with an increase in endothelial dysfunction as well as a proinflammatory switching during macrophage polarization in vitro [13,14]. We investigated the effect of Mox-LDL on essential vascular processes in endothelial cells and showed that this type of modified LDL has the potential to interfere with in vitro angiogenesis and blood vessel repair [13]. We examined the impact of Mox-LDL on inflammatory processes in macrophages and reported that Mox-LDL might be involved in aggravating the inflammatory state in macrophages by reducing their release of IL-10 [14]. Similarly, Mox-LDL was previously shown to induce the secretion of TNF-α in macrophages, enhancing inflammation, and boosting the process of atherogenesis [15]. In endothelial cells, Mox-LDL was also reported to increase IL-8 production which is considered as a crucial player during atheroma plaque progression [2,16]. On a similar note, and while exploring Mox-LDL inflammatory and proatherogenic pathways, we have recently discovered that Mox-LDL proinflammatory effects in endothelial cells may be due to the activation of the lectin-like oxidized low-density lipoprotein receptor-1 (lox-1) scavenger receptor, which is responsible for many signaling pathways that are initiated by Mox-LDL during atherogenic processes [16]. In this particular context, we were successful in completing the paradigm of a major aspect of atherosclerosis that is related to studying the effect of Mox-LDL at the level of fibrinolysis [17]. Previous clinical studies have already confirmed a hypofibrinolytic state seen in patients with atherosclerosis, and it has been proposed that pericellular fibrin deposition that is due to a decrease in endothelial cell fibrinolytic activity may negatively affect the course of the disease and enhance atheroma plaque formation by increasing the permeability of endothelial cells to LDL [18]. Meanwhile, we have reported that physiological levels of Mox-LDL have the capacity to decrease pericellular fibrinolysis in vitro in multiple cell models of atherosclerosis [19,20]. These data supported the theory that modified LDL may accelerate atherosclerosis through enhancing fibrin accumulation and deposition on the surface of endothelial cells, which could lead to an increase in inflammation and dysfunction in these cells and to a subsequent loss of the integrity of the inner lining of the blood vessel; this will culminate in an increase in its permeability to lipid infiltration, leading to foam cell formation in the subendothelial space [16]. But, it was not until very recently that we confirmed that the decrease in pro-fibrinolytic activity induced by Mox-LDL is related to the activity of the major inhibitor of fibrinolysis, neuroserpin [17]. During the process of fibrinolysis, endothelial cells are responsible for the secretion of tissue plasminogen activator (tPA), which can convert plasminogen into plasmin, facilitating the process of thrombolysis. In the nervous system, tPA activity is regulated by neuroserpin, which is a member of the serine proteinase inhibitor superfamily and the major inhibitor of tPA. In the context of stroke, tPA has been shown to have a crucial role, where it is usually used as a common agent to treat patients with acute thrombotic complications. On the other hand, it was demonstrated that neuroserpin may also have a beneficial and neuroprotective function during stroke because of its role in regulating excessive and uncontrolled tPA activity [21,22,23], Once again, we have shown that lox-1 mediated Mox-LDL signaling modulates neuroserpin activity in endothelial cells, which could have major implications that are not exclusive to the pathophysiology of ischemic strokes but are also connected to additional pathologies including Alzheimer’s disease and other mental illnesses [17]. It was first reported that neuroserpin binds to tPA in the brain of patients with Alzheimer’s disease, where it is associated with amyloid plaque formation; it is hypothesized that the amplified neuroserpin activity could result in a decrease in tPA function, which correlates with a diminished production of plasmin that may lead to a reduction in the dissolution of amyloid-beta aggregates in the brain tissue and a decline in synaptic activity and cognitive function [24,25].

As already mentioned, there might be a strong link that connects atherosclerosis, Mox-LDL as well as sleep disorders and psychiatric disorders. Actually, there is growing evidence nowadays that the latter disorders are regulated by a bi-directional causality. For instance, it was reported that sleep disorders multiply the risk of having episodes of psychiatric disorders [26]. Further, it is now becoming evident that many pharmacological agents can be commonly used for the treatment of both groups of illnesses [27,28]. Interestingly, it has been shown that sleep deprivation, which is known to correlate with an increase in MPO plasma levels and the risk of cardiovascular diseases [29,30], may aggravate numerous psychiatric disorders [31]. Accordingly, antidepressant therapy was reported to have a positive impact in resolving sleep disturbance in a lot of patients with major depressive disorder (MDD) [32]. Multiple studies have previously shown that patients with sleep disturbances, including insomnia and hypersomnia, are approximately 10 times more likely to develop MDD as compared to healthy individuals without sleep problems. Also, other studies have indicated that having insomnia at one time in life significantly raises the risk for the development of new onset MDD [33,34].

On the other hand, it was recently reported that inflammation and brain-derived neurotrophic factor (BDNF) can be considered as important risk factors for the development of MDD [35]. There is growing evidence nowadays that vascular inflammation correlates with anxiety and depression and that patients with peripheral arterial disease exhibit a higher rate of depression that might be related to ecosanoid-based vascular inflammation [36]. On a related note, it has also been hypothesized that the different isoforms of the lipoxygenase enzyme, including 5-lipoxygenase, could be involved in promoting atherosclerosis and poor mental health (anxiety, depression…) through the production of oxidized LDL and ecosanoids, respectively. However, the pathophysiological link between those illnesses remains unclear [36,37,38].

Although little is known about the link between inflammation and BDNF in the brain, emerging evidence suggests that these factors could interact in a bidirectional way in MDD, both regulating disease pathogenesis and affecting the development of depressive symptoms. It was shown that inflammatory factors may lead to a reduction in BDNF expression, and the latter may also play an opposing role in negatively regulating inflammatory processes in the brain [39,40,41,42,43,44]. BDNF is a member of the neurotrophin family of growth factors and its antidepressant activity as well as its role in MDD has been very well studied [35]. BDNF is synthesized as a precursor protein (pre-pro BDNF) in the endoplasmic reticulum and then processed into proBDNF in the Golgi apparatus after cleavage of its pre-domain, before it is either secreted in its precursor form or mature form (mBDNF); the production of the latter form of BDNF requires either intracellular or extracellular proteolytic cleavage of proBDNF [45]. mBDNF binds to TrkB receptors with high affinity, leading to receptor dimerization and autophosphorylation of tyrosine residues in its intracellular domain; this will subsequently lead to the initiation of signaling pathways that will activate multiple transcription factors, including CREB, culminating in the triggering of various mechanisms that are involved in cell survival and proliferation, synaptogenesis, memory and the inhibition of long-term depression [46]. It was shown that a lot of factors are associated with the modulation of the expression and function of BDNF. While early life inflammation and social stress may be involved in the reduction of BDNF expression, exercise was shown to have a positive effect at this level, with the potential to increase BDNF levels and to protect against the adverse effects of stressful conditions [47,48,49]. Some studies have suggested that BDNF levels may also be associated with neurodegenerative disorders such as Alzheimer’s disease as a result of an age-related decline in its activity [50,51]. For decades now, scientists have been researching BDNF as a potential biomarker for depression. Clinical studies have pointed out that altered BDNF-TrkB signaling activity is closely linked to depression [52,53]. Accordingly, it has been demonstrated that there is a negative correlation between BDNF levels and symptoms of depression, and multiple studies have reported a lower level of BDNF in depressed patients as compared to healthy controls [54,55,56]. Additional studies have shown that peripheral BDNF levels reflect BDNF expression in the central nervous system, since BDNF can cross the blood–brain barrier and serum and brain levels of BDNF positively correlate with one another [57,58]. Also, multiple studies have reported that modulating the activity of BDNF decreases depressive-like behavior in animal models of depression [59,60]. Further, it was shown that antidepressant treatments increase circulating BDNF levels in human subjects, which supports some current cellular and animal models of antidepressant effects and functions [61].

Interestingly, the tPA/plasmin system was shown to play a crucial role in the processing of proBDNF into its mature form. However, the involvement of the tPA-BDNF pathway in MDD is still not very well studied. Even so, it has recently been reported that MDD patients exhibit a lower serum level of tPA and BDNF and this level is reversed after multiple weeks of antidepressant treatment [62,63]. Although previously and conventionally recognized to be involved in thrombolytic mechanisms because of its role in the dissolution of fibrin, the tPA/plasminogen system is being rediscovered and investigated nowadays in the context of MDD [64,65]. In accordance with previous findings that link low plasma tPA levels to depression, it was equally demonstrated that plasminogen activator inhibitor-1 (PAI-1), a major inhibitor of tPA, is upregulated in both the hippocampus and prefrontal cortexes in animal models of depression as well as in the serum of human patients with symptoms of depression [65,66]. Therefore, it is speculated that a decrease in the activity of the tPA/plasminogen system that is due to a negative effect of plasminogen activator inhibitors may lead to insufficient production of mature BDNF, resulting in the impaired neural plasticity seen in the case of depression [62].

Collectively, all reports suggest that the tPA–lysis pathway may be involved in the pathogenesis of a heterogeneous branch of diseases including atherosclerosis and depression and that Mox-LDL may play a very important role at level (Figure 1). Thus, it is imperative that the scientific community takes action to reconsider Mox-LDL and the exclusive set of proteins that spans neurosepin/PAI-1, tPA and plasminogen as a biomarker panel for both atherosclerosis and MDD. Better understanding of the interactions between the latter factors within the context of atherosclerosis, cerebrovascular diseases and neuropsychiatric disorders is critical in highlighting the potentially interrelated pathological mechanisms that are responsible for the initiation and progression of both groups of illnesses. This will promote new avenues of research supporting the development of a broader perspective that could complete the paradigm of atherosclerosis and MDD, providing novel therapeutic opportunities and common treatment protocols for millions of patients worldwide.

## Figures and Tables

**Figure 1:**
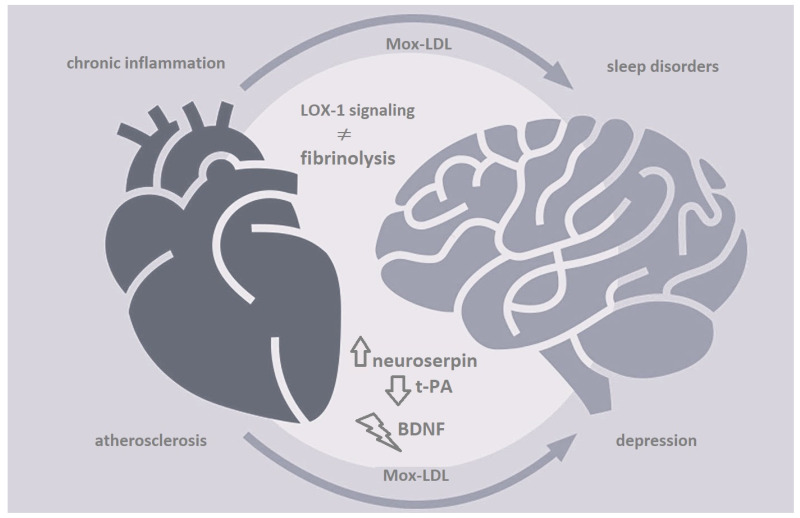
Myeloperoxidase oxidized LDL (Mox-LDL) plays a crucial role in the progression of atherosclerosis and the pathogenesis of multiple chronic inflammatory illnesses including sleep disorders. Mox-LDL is mainly involved in increasing inflammation and in regulating the process of fibrinolysis in endothelial cells during the course of the disease through lectin-like oxidized low-density lipoprotein receptor-1 (lox-1) signaling pathways and the modulation of neuroserpin activity. This would affect the processing of brain-derived neurotrophic factor (BDNF) into its mature form by altering the activity of tissue plasminogen activator (tPA) in the brain, resulting in impaired neural plasticity and depression.

## Data Availability

No new data were created or generated during the present study.
